# QAMT: An LLM-Based Framework for Quality-Assured Medical Time-Series Data Generation

**DOI:** 10.3390/s25175482

**Published:** 2025-09-03

**Authors:** Yi Luo, Yong Zhang, Chunxiao Xing, Peng Ren, Xinhao Liu

**Affiliations:** 1 School of Computer Science and Technology, Beijing Institute of Technology, Beijing 100081, China; luoyi@bit.edu.cn (Y.L.); liuxinhao@bit.edu.cn (X.L.); 2 BNRist, DCST, RIIT, Tsinghua University, Beijing 100084, China; xingcx@tsinghua.edu.cn (C.X.); renpeng@tsinghua.edu.cn (P.R.)

**Keywords:** medical time-series data generation, large language models, health knowledge graph, data quality assurance

## Abstract

The extensive deployment of diverse sensors in hospitals has resulted in the collection of various medical time-series data. However, these real-world medical time-series data suffer from limited volume, poor data quality, and privacy concerns, resulting in performance degradation in downstream tasks, such as medical research and clinical decision-making. Existing studies provide generated medical data as a supplement or alternative to real-world data. However, medical time-series data are inherently complex, including temporal data such as laboratory measurements and static event data such as demographics and clinical outcomes, with each patient’s temporal data being influenced by their static event data. This intrinsic complexity makes the generation of high-quality medical time-series data particularly challenging. Traditional methods typically employ Generative Adversarial Networks (GANs) or Variational Autoencoders (VAEs), but these methods struggle to generate high-quality static event data of medical time-series data and often lack interpretability. Currently, large language models (LLMs) introduce new opportunities for medical data generation, but they face difficulties in generating temporal data and have challenges in specific domain generation tasks. In this study, we are the first to propose an LLM-based framework for modularly generating medical time-series data, QAMT, which generates quality-assured data and ensures the interpretability of the generation process. QAMT constructs a reliable health knowledge graph to provide medical expertise to the LLMs and designs dual modules to simultaneously generate static event data and temporal data, constituting high-quality medical time-series data. Moreover, QAMT introduces a quality assurance module to evaluate the generated data. Unlike existing methods, QAMT preserves the interpretability of the data generation process. Experimental results show that QAMT can generate higher-quality time-series medical data compared with existing methods.

## 1. Introduction

With the development of Internet-of-Things (IoT) technologies, hospitals are increasingly equipped with a wide array of sensors that monitor and capture various medical data. Since these sensors record data continuously (e.g., vital signs), most of the medical data exist in the form of time-series data. These data support medical research through effective exploration and mining [1] and offer valuable assistance in tasks such as health monitoring [2], clinical decision-making [3], disease diagnosis [4,5], and treatment recommendation [6].

Although medical time-series data have demonstrated substantial value in various downstream tasks, their acquisition remains a significant bottleneck. On one hand, although sensors collect data in real time, issues such as sensor malfunctions and the inherent complexity of medical time-series data often lead to challenges in data quality. As a result, medical time-series datasets commonly used, such as eICU [7] and MIMIC-III [8], suffer from missing values, inaccuracies, incompleteness, etc., ultimately resulting in poor data quality [9]. On the other hand, because medical time-series data contain highly sensitive information (e.g., patients’ age, gender), their access is typically subject to strict privacy regulations and governance controls [10], resulting in limited data volume and privacy concerns. Some studies attempt to mitigate privacy risks by removing personal information from medical data. However, even de-identified data remain vulnerable to re-identification [11,12]. Other studies also explore the use of federated learning to address this issue. However, these works still face challenges related to system deployment and security attacks [13]. Motivated by these limitations, generated medical time-series data offer a supplement and alternative to real-world data.

However, due to the complexity of medical time-series data, which encompass a wide variety of variables that interact in complex and nonlinear ways, generating such data is challenging. A critical aspect of medical time-series data is the presence of static event data (e.g., demographics, clinical outcomes), which can significantly influence temporal data (e.g., vital signs, laboratory measurements). For instance, an in-hospital patient’s temporal data, which would include hundreds of variables, such as heart rate and blood pressure, can be influenced by several static event variables, including age, comorbidities, etc. We define an effective medical time-series generation model as one that jointly models both static event data and temporal data. Consequently, generating medical time-series data presents several significant challenges:

- **Challenge 1: Joint Generation of Temporal Data and Static Event Data.** Medical time-series data consist of diverse data types, each with unique characteristics. Among them, static event data are generally high-dimensional and discrete (e.g., demographics, clinical outcomes), whereas temporal data tend to be lower-dimensional and continuous (e.g., vital signs, laboratory measurements). Therefore, jointly generating medical time-series data that include both static and temporal components is essential for producing comprehensive and realistic datasets.
- **Challenge 2: Clinical Constraints and Variable Dependencies.** Many variables in medical time-series data are governed by clinical constraints, and their values often exhibit strong interdependencies. For example, a patient’s systolic blood pressure value of zero is clinically impossible. Additionally, if a patient consistently exhibits systolic blood pressure readings above 150 mmHg, a final diagnosis would not be hypotension. Accurately modeling these constraints and dependencies is essential to ensure the clinical plausibility of generated data.
- **Challenge 3: Need for Interpretability.** The generation process of medical time-series data should be interpretable. In clinical research and practice, it is crucial for stakeholders to understand how the data are produced in order to evaluate their quality, support downstream applications, and maintain trust in data-driven healthcare systems.

Table 1 shows that traditional medical time-series data generation methods based on deep learning techniques primarily rely on generative models, such as Variational Autoencoders (VAEs) [14] and Generative Adversarial Networks (GANs) [15]. However, although GANs and VAEs have demonstrated strong performance in generating long-sequence temporal data, they face significant challenges in simultaneously producing high-quality static event data. A few studies have attempted to address this by incorporating statistical summaries of the generated temporal data as inputs to guide the generation of static event data [16]. Nevertheless, GANs and VAEs still struggle to generate sparse one-hot encoded representations [17] with satisfactory fidelity. In addition, it is challenging to incorporate clinical constraints and variable dependencies into these models, and the black-box nature of deep learning models further limits the interpretability of the generation process. The emergence of large language models (LLMs) presents a promising new direction for medical time-series data generation [9,10]. LLMs have demonstrated strong capabilities in modeling complex distributions over discrete data [18]. However, since LLMs are primarily designed for text-based tasks, they struggle to jointly generate high-quality continuous temporal data [10]. Moreover, due to their limited exposure to domain-specific medical knowledge, generating clinically meaningful and high-quality medical time-series data remains a challenge for LLMs [19]. A recent study [20] proposes a pipeline framework for medical time-series data generation. However, this framework lacks clinical constraints and variable dependencies, as well as interpretability in the data generation process. Therefore, jointly generating high-quality medical time-series data that include temporal data and static event data with an interpretable generation process remains a significant challenge in current research.

In this study, we propose a modularized LLM-based framework for **Q**ality-**A**ssured **M**edical **T**ime-series data generation, QAMT. To incorporate domain-specific medical knowledge into LLMs and guide both the generation and quality assurance of medical time-series data, we leverage a reliable Health Knowledge Graph Builder, HKGB [30], to build a health knowledge graph (HKG). During the medical time-series generating process, QAMT adopts dual modules, combining GANs for generating temporal data and health knowledge graph-based retrieval augmented generation for generating static event data. This design leverages the advantages of existing methods and jointly generates medical time-series data. To assess the quality of the generated medical time-series data, QAMT integrates a quality assurance module that evaluates the data through clinical constraints and inter-variable dependencies, using the capabilities of the HKGB, LLMs, and a chain-of-thought prompting over HKG. In contrast to existing methods, QAMT maintains interpretability throughout the medical time-series data generation process. The main contributions of this work are as follows:

- QAMT jointly generates medical time-series data, which include both continuous temporal data and discrete static event data.
- QAMT ensures the quality assurance of the generated data by accounting for real-world clinical constraints and variable dependencies.
- QAMT enables interpretability in the medical time-series data generation process.
- QAMT is evaluated on the eICU and MIMIC-III datasets, and demonstrates superior performance compared to state-of-the-art models in terms of fidelity, utility, and privacy.

In this paper, we outline the problem definition and review related work in Section 2. Section 3 introduces the overall architecture and workflow of the proposed QAMT framework. In Section 4, we delve into the design of the health knowledge graph (HKG) module and explain the motivation for incorporating HKG. Section 5 details the dual modules responsible for jointly generating medical time-series data, including the static event data generation module and temporal data generation module. We present our data quality assurance module, including how clinical constraints and variable dependencies are enforced in Section 6. In Section 7, we provide an explanation of the interpretability of QAMT. In Section 8, we provide experimental results to evaluate our proposed QAMT. Finally, Section 9 summarizes the key contributions of this work and discusses potential directions for future research.

## 2. Preliminaries and Related Work

In this chapter, Section 2.1 provides the definition of our medical time-series data generation task. Section 2.2, Section 2.3, Section 2.4 discuss the limitations of existing work in jointly generating high-quality and interpretable medical time-series data.

### 2.1. Definition

A medical time-series dataset is denoted as $D={\left\{P_{i}\right\}}_{i=1}^{N}$, where *N* represents the number of patients, and $P_{i}$ denotes the medical time-series data of the *i*th patient. Each patient record $P_{i}$ consists of a sequence of clinical visits, denoted as $P_{i}={\left\{V_{ij},T_{ij}\right\}}_{j=1}^{|V_{i}|}$, and each visit $V_{ij}$ at time $T_{ij}$ is defined as a tuple $V_{ij}=\left\{c_{ij},y_{ij},E_{ij},t_{ij}\right\}$, where: $c_{ij}$ is a set of covariates (e.g., demographics) of this patient, $y_{ij}$ is a set of clinical outcomes (e.g., expire), $E_{ij}={\left\{e_{m}\right\}}_{m=1}^{|E_{j}|}$ is a sequence of clinical events (e.g., ICD diagnosis codes), and $t_{ij}$ is a collection of temporal data recorded in in-patient settings, including vital signs and laboratory measurements [10]. We refer to $m_{ij}=\left\{c_{ij},y_{ij},E_{ij}\right\}$ collectively as **static event data** (high-dimensional and discrete data), and $t_{ij}$ as **temporal data** (low-dimensional and continuous data). Each temporal data element $t_{ij}$ is further represented as $t_{ij}={\left\{time_{k},{\left\{(n_{kp},x_{kp})\right\}}_{p=1}^{|L_{k}|}\right\}}_{k=1}^{|t_{ij}|}$, where $|t_{ij}|$ denotes the total number of measurement time points, $|L_{k}|$ denotes the number of variables measured at time point $time_{k}$, $n_{kp}$ is the name of the *p*th variable measured at time $time_{k}$, and $x_{kp}$ is the corresponding observed value. In this paper, our task is to jointly generate high-quality and interpretable medical time-series data, including both static event data and temporal data.

### 2.2. Medical Time-Series Data Generation

Due to the complexity of medical time-series data, as shown in Table 1, few studies have addressed the joint generation of medical time-series data.

A considerable amount of research has focused on the generation of a sequence of static event data. Traditional methods, such as GAN-based methods [20,21] and VAE-based methods [23], have been widely used. However, the discrete and high-dimensional nature of static event data makes it challenging for them to guarantee the quality of the generated data. Recently, LLMs have been explored for static event data generation [19,24]. Empirical studies have demonstrated that LLMs outperform GAN- and VAE-based methods in static event data generation [31,32], largely due to their superior capabilities in semantic reasoning and contextual understanding.

Other research on temporal data generation has primarily focused on producing data with meaningful patterns and trends. Traditional generative techniques, such as GANs [25,26,27] and VAEs [28], have demonstrated strong potential for generating temporal data. Existing studies have shown that GAN-based methods generally outperform VAE-based methods in generating temporal data [33]. The application of LLMs to temporal data generation remains relatively underexplored [10]. This limitation is due to LLMs being inherently designed for text-based tasks, and they must treat numerical temporal data as sequences of tokens.

Few studies can jointly generate both static event data and temporal data. Traditional GAN-based methods [16] generate medical time-series data by a hybrid multi-generator framework. LLM-based methods, such as HALO [9] and SynEHRgy [10], employ novel tokenization strategies. Although these methods achieve joint generation of medical time-series data, they face notable limitations: GAN-based methods often produce unreliable static event data, while LLM-based methods struggle to generate high-quality temporal data and face high computational cost. Therefore, modularizing the medical time-series data generation task using two modules to generate static event and temporal data may yield better performance than relying on a single model.

### 2.3. Medical Time-Series Data Quality Assurance

Data quality assurance plays a crucial role in guaranteeing the quality of generated medical time-series data. This assurance must consider not only clinical constraints (value range limits on temporal variables and logical limits among sequential diagnoses in static event variables) but also variable dependencies (logical inferences exist between different variables). In the following, we provide an overview of existing data assurance methods.

A few existing studies have addressed clinical constraints in medical time-series data generation. As shown in Table 1, some methods perform pre-generation quality assurance by incorporating quality assurance mechanisms directly within the generative model architecture [16,24] or leveraging the reasoning capabilities of LLMs to ensure logical consistency [24]. Other methods [34] perform post-generation quality assurance by employing sampling and data transformation techniques to maintain value ranges and data integrity. Although these methods can enforce numerical value ranges for temporal data, they fall short in validating static event data, which require logical constraints. For example, a patient cannot have a diagnosis of poliomyelitis immediately followed by Alzheimer’s.

Due to the complex logical relationships among variables in medical time-series data, very few studies have addressed the assurance of variable dependencies in generated data. Existing work, such as PromptEHR [19], leverages the reasoning capabilities of LLMs to infer dependencies from one variable to another during the generation process. However, the generation process of LLMs is prone to hallucination [35], which limits the reliability of such inferred dependencies. As a result, though LLMs may capture dependencies among variables when generating data, it is also essential to validate the variable dependencies of the generated medical time-series data again by post-generation assurance.

### 2.4. Interpretability of Data Generation

Traditional medical time-series data generation methods, such as GANs [20,21,22,25,26] and VAEs [23,28], are inherently black-box models, making it difficult to provide meaningful explanations for the data generation process. Moreover, existing LLM-based methods [9,10,19,24] also lack the interpretability of data generation. However, the interpretability of medical time-series data generation is essential for researchers, making the interpretability of the data generation process crucial.

## 3. QAMT Overview

**Figure 1:**
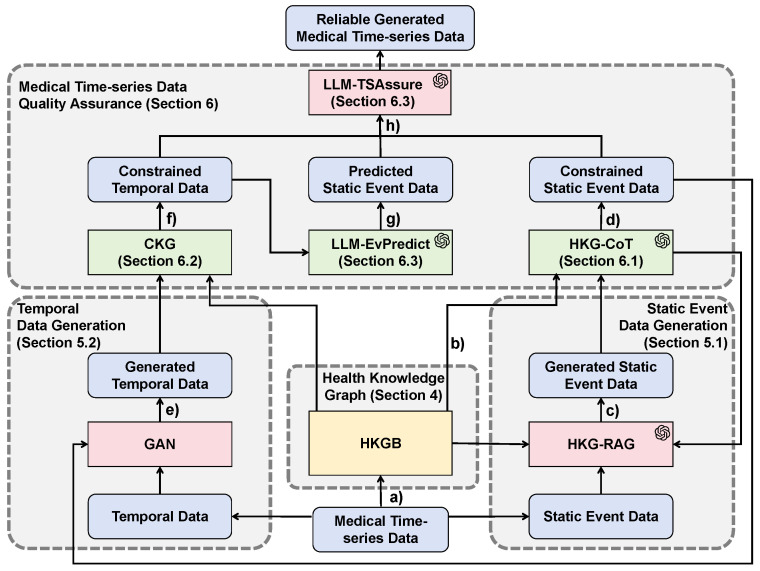
The framework of QAMT, where the blue rounded rectangles represent data, red rectangles represent generative models, green rectangles represent medical time-series data quality assurance models, and yellow rectangles represent the health knowledge graphs. The parts marked with icons indicate the use of LLMs.

QAMT enables the joint generation of high-quality medical time-series data while preserving the interpretability throughout the generation process. As illustrated in Figure 1, this framework consists of four main modules. The health knowledge graph module provides domain-specific knowledge to LLMs. The GAN-based temporal data generation module and the LLM-based static event data generation module are responsible for jointly generating medical time-series data. The medical time-series data quality assurance module evaluates the generated data to ensure the overall quality and clinical plausibility of the final generated data. The entire medical time-series data generation and quality assurance process can be divided into the following steps:

(a) Based on real-world medical time-series datasets and open knowledge bases, a health knowledge graph (HKG) is constructed using an existing Health Knowledge Graph Builder (HKGB). This knowledge graph serves as a domain-specific knowledge resource for downstream LLMs (Section 4.1).
(b) Construct HKG-CoT, a chain-of-thought (CoT) reasoning process enriched with clinical knowledge from the HKG, which provides healthcare-specific inference capabilities (Section 4.2).
(c) The static event data generation module uses Retrieval-Augmented Generation (RAG) guided by the HKG to generate static event data $m^{\prime }$ (Section 5.1), referred to as Health Knowledge Graph-based Retrieval Augmented Generation (HKG-RAG):
  (1) $$m^{\prime }=\mathrm{HKG}\text{-}\mathrm{RAG}\left(m,HKG\right)$$
(d) The medical time-series data quality assurance module then evaluates the generated static event data using HKG-CoT (Section 6.1), obtaining constrained static event data $m^{\prime \prime }$ with clinical constraints and logical consistency. Then, the evaluation results are fed back to the static event data generation module:
  (2) $$\mathrm{Constraint}\;1:\;CC_{1}\left(m^{\prime }\right)=\mathrm{HKG}\text{-}\mathrm{CoT}\left(m^{\prime },HKG\right)\in \left\{0,1\right\}$$
  (3) $$m^{\prime \prime }=CC_{1}\left(m^{\prime }\right)\cdot m^{\prime }$$
(e) The temporal data generation module uses a GAN to generate temporal data $t^{\prime }$ (Section 5.2), leveraging constrained static event data as conditional guidance:
  (4) $$t^{\prime }=\mathrm{GAN}\left(t,m^{\prime \prime }\right)$$
(f) The generated temporal data are further validated by the medical time-series data quality assurance module, using the Concept Knowledge Graph (CKG), which is a subgraph within the HKG (Section 6.2), to check for clinical value range constraints and plausibility and obtain constrained temporal data:
  (5) $$\mathrm{Constraint}\;2:\;CC_{2}\left(t^{\prime }\right)=\mathrm{CKG}\left(t^{\prime },HKG\right)\in \left\{0,1\right\}$$
(g) The constrained temporal data are then input to an LLM-based diagnostic model, LLM-EvPredict, which predicts its corresponding static event data. Then, the medical time-series data quality assurance module compares the predicted static event data with the previously constrained static event data using the LLM-TSAssure (Section 6.3):
  (6) $$\mathrm{Constraint}\;3:\;VD\left(m^{\prime },t^{\prime }\right)=\mathrm{LLM}\text{-}\mathrm{TSAssure}\left(m^{\prime },\mathrm{LLM}\text{-}\mathrm{EvPredict}\left(t^{\prime }\right)\right)\in \left\{0,1\right\}$$
(h) Finally, if the predicted static event data and constrained static event data are deemed consistent, the static event data and temporal data are considered to satisfy variable dependencies and are jointly assembled into final, reliable synthetic medical time-series data $V^{\prime }$ (Section 6.3):
  (7) $$V^{\prime }=VD\left(m^{\prime },t^{\prime }\right)\left(CC_{1}\left(m^{\prime }\right)\cdot m^{\prime }⊕CC_{2}\left(t^{\prime }\right)\cdot t^{\prime }\right)$$

The following sections provide a detailed description of each module and explain how they contribute to the joint generation and quality assurance of medical time-series data.

## 4. Health Knowledge Graph Module

The emergence of LLMs has introduced new potential for data generation. However, existing LLMs lack sufficient domain-specific knowledge in the medical time-series data generation task, leading them to generate hallucinated information. One solution involves pretraining LLMs on domain-specific corpora to enhance their medical knowledge [36,37]. However, it is computationally expensive. Another strategy involves prompt tuning to improve LLM performance [37,38], which requires relevant domain knowledge. Therefore, an increasingly explored method is to integrate knowledge graphs (KGs) with LLMs [39,40,41,42,43]. By leveraging the structured domain knowledge encoded in KGs, LLMs can be guided toward more accurate generation and reasoning. In this paper, we construct a health knowledge graph module to provide domain-specific expertise to the LLMs. In Section 4.1, we describe how we construct the HKG. In Section 4.2, we explain how HKG is used to guide reliable CoT reasoning. In Section 4.3, we demonstrate how HKG assists in generating trustworthy medical information.

### 4.1. HKG

**Figure 2:**
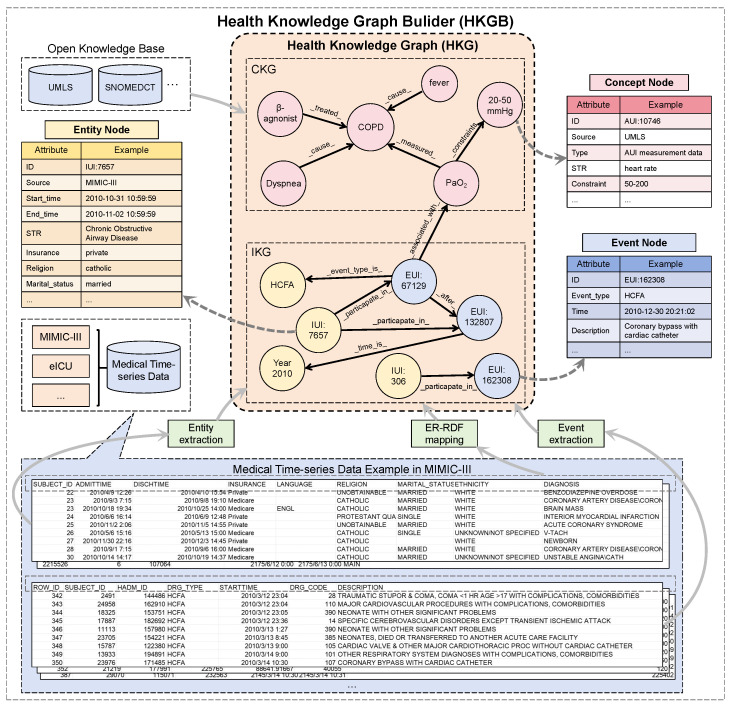
The detailed workflow of the HKGB to construct an HKG.

To construct a reliable HKG, we leverage an existing HKGB [30]. As shown in Figure 2, the HKGB is an end-to-end platform designed to construct disease-specific and scalable health knowledge graphs (HKGs) from diverse sources as well as evaluate its generated HKG. In this study, we built a reliable HKG by leveraging external knowledge bases together with medical time-series data (e.g., MIMIC-III and eICU). The HKG constructed by the HKGB consists of three types of nodes: concept nodes, entity nodes, and event nodes. Among them, concept nodes are extracted from open knowledge bases and include medical information such as diseases, symptoms, drugs, treatments, clinical indicators, and clinical constraints. We refer to the subgraph containing only concept nodes as the Concept Knowledge Graph (CKG). Since the construction of the CKG relies on external knowledge bases, its update is relatively slow.

In contrast, entity nodes and event nodes are derived from real medical time-series datasets. Entity nodes, which represent patient information (e.g., demographic) or contextual information, are obtained through event extraction, and event nodes, which capture static clinical events in the medical time-series data, are extracted through entity extraction. Furthermore, we use ER-RDF to extract column-level information from the datasets to enrich the node information. We refer to the subgraph containing entity and event nodes as the Instance Knowledge Graph (IKG). Since the construction of the IKG depends on real medical time-series datasets, it is frequently updated and continuously expanded as new medical time-series data become available.

In this work, the HKG is defined as the integration of the CKG and IKG, thereby capturing both general medical knowledge and instance-level clinical information. Given the complexity and heterogeneity of these nodes, the HKG defines edges to represent relationships between them, with several illustrative examples provided in Table 2.

### 4.2. HKG-CoT

Handling reasoning tasks such as arithmetic, commonsense, and symbolic reasoning has always been a challenge [44]. Previous studies have explored the use of KGs for logical reasoning [45]. However, selecting nodes based solely on similarity does not necessarily lead to correct or complete reasoning outcomes. With the rapid development of LLMs, recent work has explored LLMs’ potential in reasoning by constructing a CoT [38], which aims to solve complex reasoning problems by generating a sequence of intermediate reasoning steps through manually designed prompts. Although CoT prompting has demonstrated promising performance in reasoning, it often suffers from hallucinations when applied to knowledge-intensive tasks, primarily due to the lack of related knowledge. To address this limitation, recent work proposed the concept of KG-CoT [41], which combines the explicit relational structure of KGs with the step-by-step reasoning capabilities of CoT prompting. This method decomposes complex problem-solving into manageable steps, enhancing the reasoning capabilities of LLMs while providing an observable reasoning process that improves interpretability [35]. However, its multi-turn question mechanism inevitably leads to increased computational cost [46]. Since the generated static event data consist of various events for multiple patients, and there should be certain logical constraints among the events of each individual, it is reasonable to use a CoT to infer the logic between a patient’s multiple events. Therefore, in this paper, we construct an HKG-CoT to fulfill static event data quality assurance.

### 4.3. HKG-RAG

To address the limitations of LLMs in accessing external domain-specific knowledge, many studies have adopted Retrieval-Augmented Generation (RAG) [47], which enhances LLMs by incorporating few-shot prompts retrieved from external sources (e.g., real-world medical time-series data or open knowledge bases). However, information retrieved directly from data-rich databases often lacks reliability and interpretability [48]. Recent research has therefore focused on integrating KGs into retrieval strategies to strengthen LLMs’ generation ability [49,50]. Compared to databases, KGs provide structured and inferable knowledge, making them more suitable for enhancing RAG. Recent work proposed KG-RAG [40], demonstrating that KGs could effectively enhance the performance of LLMs. In the generation tasks, RAG has the advantage of encoding sensitive data in a secure manner, thereby reducing the risk of privacy leakage. Furthermore, compared to a CoT, RAG can eliminate certain intermediate generation steps, thus lowering computational costs. Given that static event data generation requires the rapid synthesis of large volumes of data while minimizing privacy risks during the generation process, we propose a method, HKG-RAG, to support the generation of static event data.

## 5. Medical Time-Series Data Generation Module

As analyzed in Section 2.2, since GANs and LLMs each have their advantages in static event data generation and temporal data generation, respectively, QAMT adopts dual modules to accomplish joint medical time-series data generation: the LLM-based static event data generation module (Section 5.1) and the GAN-based temporal data generation module (Section 5.2).

### 5.1. Static Event Data Generation Module

**Figure 3:**
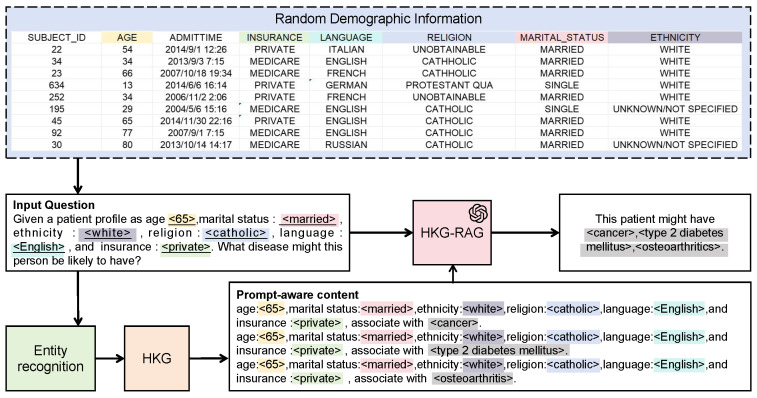
HKG-RAG workflow. The demographic information is randomly generated. An LLM is then used to assess its validity to avoid logically inconsistent cases, such as an age of “6” with a marital status of “MARRIED”. Only demographic data that pass this logical check are used as input for generating complete static event data.

In the static event data generation module, we employ the HKG-RAG introduced in Section 4.3. As illustrated in Figure 3, the process of static event data generation consists of the following steps:

**Step 1: Privacy-insensitive demographic sampling and prompt customization.** Demographic information *c*, such as patient ID, age, diagnosis time, religion, and marital status, is randomly sampled from real-world medical time-series data. This information is first checked by an LLM, obtaining reliable demographic information $c^{\prime }$ as shown in Equation (8), and then used to construct a customized prompt *q*, which is fed into another LLM:

(8) $$c^{\prime }=\mathrm{LLM}\text{-}\mathrm{judge}\left(Sample\left(c\right)\right)$$

**Step 2: Entity recognition.** Entities $e=\left\{e_{n1},e_{n2}\ldots e_{nm}\right\}\subseteq q$ are extracted from the input prompt $q=NL\left(c^{\prime }\right)$ and matched to corresponding nodes in the HKG:

(9) $$N_{q}=\left\{n|sim\left(n,e_{ni}\right)>\tau \right\}\subseteq \mathrm{HKG}$$

where τ is the threshold value. During the entity extraction, QAMT employs zero-shot prompting on an LLM distinct from the previous one to ensure accuracy. Next, entity linking is performed to obtain the corresponding entities in the HKG. We utilize the sentence embedding model “all-MiniLM-L6-v2” to encode entity nodes into dense vector representations [51]. The similarity between the extracted entities and HKG nodes is then calculated in this embedding space to determine the best match.

**Step 3: Contextual retrieval of clinical outcomes and events based on the HKG.** In the HKG, entity nodes are connected to event nodes through contextual triples (Subject, Predicate, Object), following a defined schema as shown in Table 2. Since multiple entities may be extracted from one prompt and may correspond to several entity nodes in the HKG, we select the top-K*_rag_* most frequently occurring objects across inferred triples as the final context targets. These are then structured into n-ary tuples: (Subject_1_, Subject_2_, …, Subject*_n_*, Predicate, Object). These tuples can be directly transformed into a natural language sentence using the following pattern: (Subject_1_, Subject_2_, …, Subject*_n_*, Predicate, Object) → Subjects predicateName Object.

**Step 4: Generation of clinical outcomes and events.** The prompt-aware content is used as few-shot prompts for the LLM to generate the final output. The generated clinical outcomes and events are then matched with the corresponding demographic information to form the final generated static event data.

(10) $$m^{\prime }=\tilde{m_{S}}⊕\mathrm{HKG}\text{-}\mathrm{RAG}\left(\tilde{m_{S}},\;fewshot\right).$$

**Step 5: Feedback mechanism.** In the initial generation process, we set the size of top-K*rag* to 5. Subsequently, the medical time-series data quality assurance module evaluates the generated static event data (Section 6.1). If the data generated by HKG-RAG fail to satisfy the clinical constraints, we expand top-K*rag* to 10, enabling the generation of more diverse results and correcting previously erroneous outputs. By feeding back the evaluation results from the time-series data quality assurance module to the static event data generation module, the efficiency of data generation can be improved.

### 5.2. Temporal Data Generation Module

In the temporal data generation module, we adopt a GAN, which has demonstrated strong performance in continuous data generation. To establish a connection between it and the static event data generation module, we incorporate the statistical information of constrained static event data m′ after validation (Section 6.1) as input to assist the generation of temporal data [52]:

(11) $$\min_{G}\max_{D}E_{t∼P_{\mathrm{real}\;}}\left[\log D\left(t|\theta_{D}\right)\right]+E_{z∼P_{z}}\left[\log\left(1-D\left(G\left(z,m^{\prime }|\theta_{G}\right)\right)\right)\right]$$

In this GAN model, we introduce an attention mechanism to mitigate the impact of noise. Additionally, we incorporate learned positional encoding to integrate position information into the attention computation process, preserving relative distance information within the sequence.

The generator accepts input random noise vectors *z* and statistical information derived from the static event data *c*, generating time-series data $D_{T}=G_{t}\left(z,c;\theta_{g}\right)$, which captures variable dependencies between the static event data and temporal data.

## 6. Medical Time-Series Data Quality Assurance Module

Real-world medical time-series data often exhibit multiple constraints, including logical constraints within a single patient’s static event data (e.g., a patient diagnosed with poliomyelitis is unlikely to be diagnosed with Alzheimer’s disease in a short time) and value constraints on temporal data (e.g., heart rate values cannot be zero). Moreover, there are typically variable dependencies among variables (e.g., patients with high systolic blood pressure are more likely to have hypertension).

To ensure higher quality in the generated medical time-series data, we apply the HKG-CoT (Section 6.1) and CKG (Section 6.2) to enforce constraints on the outputs from the static event data and the temporal data generation module, respectively. In addition, we use LLMs to predict the static event data corresponding to the constrained temporal data. By comparing the predicted static event data with the constrained static event data, we validate the variable dependencies in the generated medical time-series data (Section 6.3).

### 6.1. Clinical Constraint Assurance in Static Event Data

**Figure 4:**
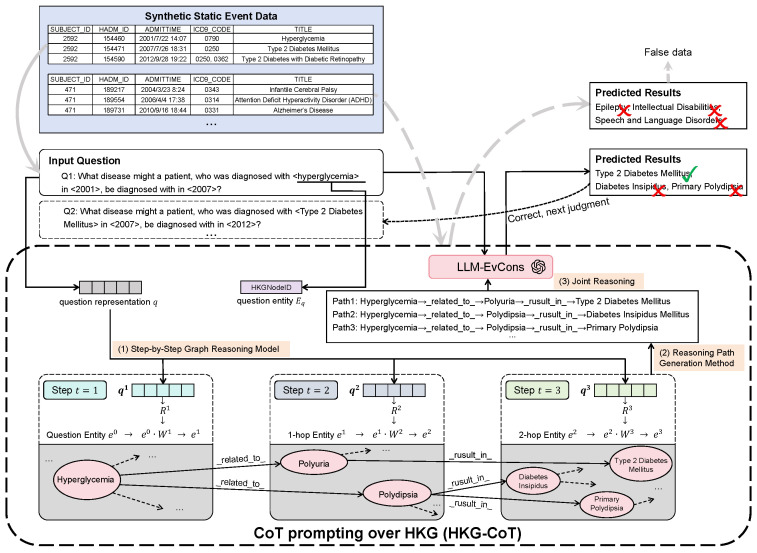
HKG-CoT workflow. It presents an example of how the HKG-CoT is used for static event data quality assurance. The solid lines indicate that the first event in the generated data is extracted and inserted into a customized prompt, which is then submitted to the LLM for reasoning. The HKG-CoT generates a response Type 2 Diabetes Mellitus, Diabetes Insipidus, Primary Polydipsia for the question. Since Type 2 Diabetes Mellitus matches the second event in the data sequence, showing as a check mark, the first event passes the validation, and the process continues by questioning the next event, and so on. If all events pass this validation, the entire dataset is considered trustworthy. In contrast, as shown by the dashed lines, if the LLM’s response does not match the event, showing as a cross mark, and does not contain the next event, the event sequence is deemed invalid and discarded.

Since static event data often exhibit inherent logical relationships, we apply the HKG-CoT introduced in Section 4.2 to enforce logical constraints on the generated static event data. Figure 4 illustrates the detailed process of the HKG-CoT:

**Step 1: Step-by-step graph reasoning model.** Let *n* denote the number of entities in the HKG. We first initialize an entity state $e^{0}\in {\left[0,1\right]}^{n}$. If the *i*th entity is mentioned in the question, $e_{i}^{0}\in e^{0}$ is initialized to 1; otherwise, it is set to 0. The question is transformed into a one-dimensional vector *q* through embedding. Divide the graph reasoning process into *T* steps, and through the design of an attention-related function $f^{t}\left(\right)$, obtain the question representation $q^{t}=f^{t}\left(q\right)$ at step *t*, t=1,2,…,T. The question representations focus on different parts of the question context at different steps. Then, we can obtain the scores of all relations in the HKG at step *t* by using a multi-layer perception (MLP). We define a transition matrix $W^{t}$ and the update formula for *e* is $e^{t}=e^{t-1}W^{t}$. After *T* steps of reasoning, we can obtain the confidence score of each entity based on $e^{k}$.

**Step 2: Reasoning path generation method.** Based on the results of the step-by-step graph reasoning model, obtain the *k* entities with the highest confidence, denoted as $E^{k}$. Perform *T* steps of reasoning, and for each reasoning path, calculate its score based on rules. Take the entity in question as the initial entity. At step *t*, select the top-*k* intermediate reasoning paths based on the scores and add these paths to the candidate paths. After *T* steps of reasoning, select the top-*k* candidate reasoning paths with the highest scores to form the final reasoning paths: $\left({\mathrm{Path}}_{1},\;{\mathrm{Path}}_{2},\;\ldots ,\;{\mathrm{Path}}_{k}\right)$.

**Step 3: Reasoning.** Serialize the selected *k* final reasoning paths and use detailed instructions to prompt the LLM to generate answers using these reasoning paths. Since generated static event data consist of multiple events of a single patient, we form a question by combining the first event (the event data are sorted by timestamp) with a pre-defined prompt. If the answer produced by the HKG-CoT does not contain the entity of the second event, we consider the patient’s event data to be incorrect. If this is the first time the data undergo validation, the validation results are fed back to the static event generation module for correction (Section 5.1). If the data remain incorrect even after feedback-based revision, they are discarded. Conversely, if the event data are deemed correct, we consider the first event as passed and continue to ask questions about the second event until the set of event data is discarded or all events of this patient are passed. After the data quality assurance conducted by the HKG-CoT, we consider the generated event data to be constrained.

### 6.2. Clinical Constraint Assurance in Temporal Data

Temporal data are often subject to value constraints. However, due to the structural complexity of medical time-series data, different datasets may record different variables, making it difficult to manually define value constraints for each variable. The CKG within the HKG introduced in Section 4.1 captures clinical constraints associated with various clinical indicators and serves as a reliable and comprehensive knowledge base. Therefore, QAMT leverages the CKG to apply value constraints to the temporal data, thereby enabling data quality assurance.

For the generated temporal data, we extract each variable and perform similarity matching with the clinical indicator nodes (concept nodes) in the CKG. By leveraging the relationships between clinical indicators and clinical constraints, we obtain the corresponding value constraints for each variable. If a variable in the temporal data exceeds its specified value range, the data are considered unreliable. Otherwise, if all values fall within the range, the data are constrained.

### 6.3. Assurance of Variable Dependencies

Due to the complex relationships among variables in medical time-series data, it is necessary to verify the dependencies between temporal data and static event data. For example, suppose a patient’s temporal data show elevated systolic blood pressure (within the acceptable value range), but the corresponding static event data indicate a diagnosis of hypotension. This inconsistency suggests that the generated medical time-series data are unreliable. Given the diagnostic relationship between temporal data and static event data, we utilize an LLM to predict diagnostic outcomes in the static event data based on the constrained temporal data. If the predicted static event data are similar to the originally constrained static event data, the generated medical time-series data are considered reliable.

Previous studies have shown that with carefully designed prompts, LLMs can successfully predict static event data from temporal inputs [53,54]. Therefore, we designed LLM-EvPredict, a static event prediction model based on temporal data. This LLM formats a patient’s temporal data into queries by organizing the variables into tuples (variable: values)*_n_*, and construct the corresponding prompt as: $\mathrm{Prompt}\;=\;{\mathrm{Instruction}}_{start}\;+\;\mathrm{Context}\;+\;{\mathrm{Instruction}}_{end}$.

The results obtained by LLM-EvPredict are written in the form of event tuples (${\mathrm{PreEvent}}_{1}$, ${\mathrm{PreEvent}}_{2}$, …, ${\mathrm{PreEvent}}_{n}$). Another model, LLM-TSAssure, is then used to compare the predicted static event data (in tuple form) with the constrained static event data of the corresponding patient (also in tuple form). If LLM-TSAssure determines that the two sets of static events are likely to come from the same patient, we consider that variable dependencies exist between the generated temporal data and static event data and retain the data. Otherwise, the data are discarded.

## 7. The Interpretability of Medical Time-Series Data Generation

Due to the high modularity of QAMT and its multiple usage of LLMs throughout the time-series data generation and quality assurance, the framework offers an interpretable generation pipeline.

**(1) Clearly defined modularization.** As shown in Figure 1, QAMT consists of four modules, each functioning independently. Specifically, steps such as (c) static event data generation, (d) static event data quality assurance, (g) static event data prediction, and (h) assurance of variable dependencies involve the formulation of question prompts and the use of LLMs to produce outputs. These interactions with the LLMs, comprising formulated questions and corresponding answers, reflect the logical flow of medical time-series data generation, thereby providing QAMT’s interpretability.

**Figure 5:**
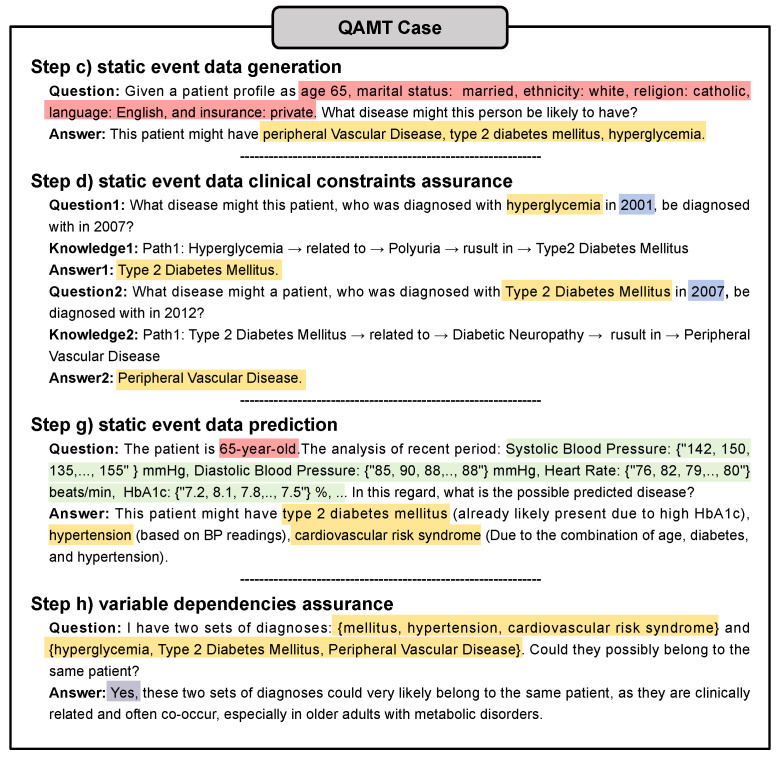
Interpretable prompt example, where (c), (d), (g) and (h) corresponds to the step introduced in Section 3.

**(2) Clear collaboration between modules.** Figure 5 illustrates the process of generating and validating static event data based on randomly sampled demographic information. The output of each step serves as input for the next, and the question–answer pairs at each step make the generation process interpretable, demonstrating the collaboration of the data generation task. Moreover, some steps, such as the clinical constraint assurance in static event data based on the HKG-CoT, further enhance interpretability by providing insight into the reasoning steps within the step itself.

## 8. Experimental Results

### 8.1. Experimental Setup

#### 8.1.1. Datasets

We conducted experiments using the MIMIC-III [8] and eICU [7] datasets. MIMIC-III is a large, publicly available database that contains a wide range of medical time-series data. In this study, we extracted 40 thousand data samples following the methodology adopted in previous work [10]. Specifically, we employed the preprocessing pipeline in [55] to extract relevant data. Except for patient demographic information (age and gender), we selected 25 phenotype labels as static event data for each visit. Furthermore, we included 41 continuous temporal variables derived from vital signs and laboratory measurements. The eICU Collaborative Research Database is a comprehensive and publicly available resource that contains data on approximately 200,000 hospitalized patients. In this study, similar to previous work [52], we extracted 13 thousand data samples. In addition to patient demographic information (age, gender, and ethnicity), we selected seven phenotype labels as static event variables and 40 continuous vital signs and laboratory measurements as temporal variables.

#### 8.1.2. Evaluation Metrics

We evaluated the generated time-series data based on fidelity, utility, and privacy [10,52].

**Fidelity.** For static event data, we assessed fidelity using the probabilities of unigram, bigram, and trigram within each visit, as well as the probabilities of sequential bigram between continuous visits. For example, the probability of a continuous visit as $\left[icd_{13},icd_{920}\right]$ was computed by dividing its frequency by the total number of patients. We then calculated the Pearson Correlation between the top 1000 *n*-gram probabilities in the real and generated datasets to evaluate the similarity in their distributions.

For temporal data, we first constructed embeddings for each patient by calculating the statistical features of their first 48 h of temporal data (minimum, maximum, mean, and standard deviation). Using these embeddings, we evaluated the fidelity by calculating the precision, recall, density, and coverage (PRDC) between the embeddings of the generated data and real data. Furthermore, we compared the correlation matrices of the real and generated data and report the mean squared error ($MSE_{corr}$) as overall correlation fidelity.

**Utility.** We assessed the utility of the generated data by evaluating their performance across four downstream tasks involving two disease types: sepsis clustering [56], sepsis treatment strategy modeling [57], ARDS (Acute Respiratory Distress Syndrome) prediction [58], and ARDS treatment strategy modeling [59]. A smaller difference between the generated data and real data in downstream tasks indicates more similarity. For the sepsis clustering task, the study evaluated the results before and after applying its proposed method using the Sum of Squares Error (SSE) metric. Accordingly, we adopted the difference in SSE improvement between real and generated data as our evaluation metric. For the sepsis treatment strategy task, we measured the difference in patient condition improvement ΔQ between models trained on generated data and real data. For ARDS prediction, we compared the AUROC scores of classifiers trained on real data and generated data within a 12 h window before onset. For ARDS treatment strategy modeling, we compared the average reduction in mortality achieved by reinforcement learning algorithms when trained on real or generated data.
**Privacy.** We adopted the Membership Inference Attack (MIA) as the evaluation metric of privacy to determine whether specific data points were included in the data [27]. We fit a K-Nearest Neighbors (KNN) model on the generated data and the real dataset and calculated their nearest distances for each patient. A significant disparity between the distance distributions in the generated and real sets indicates lower privacy. We used the Hamming distance for static event sequences and the Euclidean distance for temporal embeddings. We then fit Gaussian distributions to these distances and assess the differences between the two distributions using the Wasserstein Distance (WD), Jensen–Shannon Divergence (JSD), and Area Under the Receiver Operating Characteristic (AUROC) metrics.

#### 8.1.3. Baselines

Since QAMT is capable of jointly generating medical time-series data, we compared our method, based on Table 1, with HGAN [16], HALO [9], SynEHRgy [10], and SynTEG [20].

#### 8.1.4. Experimental Details

Since QAMT utilizes LLMs across multiple steps, including static event data generation, clinical constraint assurance in static event data, static event data prediction, and assurance of variable dependencies, we employed different models for different steps, including LLaMA 2 [60], GPT-3.5, Gemini [61], and GPT-4 [62]. The experiments were conducted in an environment equipped with an NVIDIA RTX A5000 GPU (Santa Clara, CA, USA).

### 8.2. Medical Time-Series Data Fidelity Evaluation

Table 3 presents the correlation values of *n*-gram probabilities between real and generated static event data. On the MIMIC-III dataset, although SynEHRgy achieved the best performance on trigram probabilities, our method consistently outperformed all baselines, particularly in unigram, bigram, and sequential bigram probabilities. Similarly, on the eICU dataset, our method demonstrated superior performance in unigram, trigram, and sequential bigram probabilities. These results confirm that our method is capable of generating high-quality static event data.

Table 4 reports the PRDC metrics, along with the correlation difference ($MSE_{corr}$) for evaluating the fidelity of temporal data. We found that our proposed framework achieved either the best or second-best performance in the PRDC metrics compared to the baselines. This is attributed to our modular design, which incorporates a GAN model better suited for temporal data generation. Moreover, our model achieved the lowest $MSE_{corr}$, indicating that QAMT excels in capturing variable-level correlations, thanks to the variable dependencies described in Section 5.2 and Section 6.

### 8.3. Medical Time-Series Data Utility Evaluation

Table 5 presents the performance of different models across various downstream tasks. We found that our proposed method achieved the best performance on all tasks in the MIMIC-III dataset. On the eICU dataset, our model performed best on the SepsisClustering and SepsisTreatment tasks. Compared with the MIMIC-III dataset, we included three demographic variables (static event variables) in the eICU dataset. Although these variables were also subject to quality assurance, they were randomly generated and thus carried uncertainty. As a result, our model showed some instability in the ARDSPrediction and ARDSTreatment tasks of eICU.

In addition, we applied QAMT to generate sepsis time-series data based on the real data provided by the Emergency Department of Peking University People’s Hospital. First, the generated data improved the sepsis prediction model’s accuracy as the training data [57], which assisted doctors in clinical decisions. Second, the generated data supported clinical research on sepsis, particularly in sepsis subphenotypes, revealing significant heterogeneity in inflammatory biomarkers, treatments, and consistency across cohorts [63]. Due to privacy considerations, the related medical data cannot be publicly released.

### 8.4. Medical Time-Series Data Privacy Evaluation

MIA metrics are reported in Table 6. Here, we computed privacy metrics by using the Hamming distance of static event data and the Euclidean distance of temporal data. We found that none of the methods showed a privacy risk.

### 8.5. Robustness Analysis

#### 8.5.1. Statistical Significance Test

To ensure the statistical reliability of the experimental conclusions, we repeated several experiments using the MIMIC-III dataset. For the static event data, we selected the probability of unigram and bigram in each visit as the evaluation metrics. For temporal data, we chose precision and recall as evaluation metrics. For time-series data, we selected the performance in the downstream tasks of sepsis clustering and sepsis treatment as the evaluation index. We measured the mean of the results and their 95% confidence intervals. The results are shown in Table 7. We found that the experimental results fell in a certain interval with high probability, and the worst value of that interval was still better than the vast majority of baseline methods. Therefore, our experimental conclusions are statistically significant.

#### 8.5.2. Noise Robustness Analysis

We injected 0% to 20% Gaussian noise into MIMIC-III time-series data and used the fidelity of temporal data as the evaluation metric to evaluate the noise robustness of QAMT. The results are shown in Table 8. We found that QAMT was less affected by noise, as its quality assurance module performed quality validations on the generated data. As a result, QAMT demonstrates robustness against noise.

### 8.6. Parameter Sensitivity Analysis

We also tested the sensitivity of QAMT by varying *k*, which is the number of inference paths selected in the KG-CoT (Section 5.1). The results are shown in Table 9. We found that the improvement of the fidelity of temporal data became smaller as *k* increased. Moreover, when *k* > 7, the fidelity of temporal data was no longer significantly improved by increasing *k*.

### 8.7. Ablation Experiments

To validate the importance of each module in the proposed QAMT framework, we conducted an ablation study. Specifically, QAMT_0_ only used a GAN to generate all static event data and temporal data, QAMT_1_ only used an LLM to generate all static event data and temporal data, and QAMT_2_ used a GAN to generate temporal data and an LLM to generate static event data. On the basis of QAMT_2_, QAMT_3_ added CoT prompting to ensure clinical constraints to the generated static event data. QAMT_4_ introduced the external knowledge graph HKG based on QAMT_3_. We compared the full QAMT against QAMT_0_, QAMT_1_, QAMT_2_, QAMT_3_, and QAMT_4_ across fidelity, utility, and privacy metrics on the MIMIC-III dataset to demonstrate the contribution and necessity of each module in the framework.

#### 8.7.1. Fidelity Evaluation

**Figure 6:**
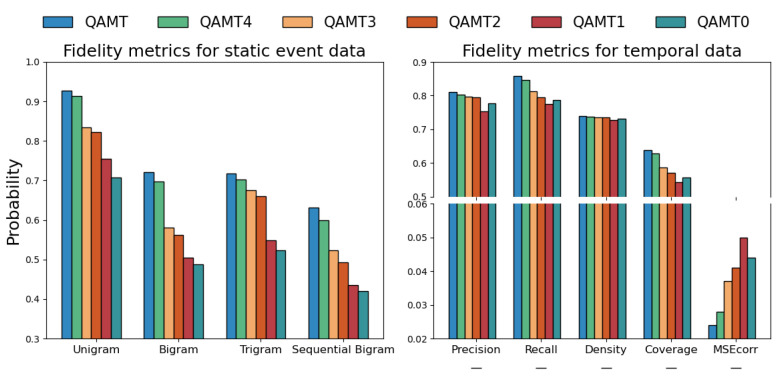
Fidelity metrics for medical time-series data in ablation experiments. Static event data results are on the left, and temporal data results are on the right.

As shown in Figure 6, from QAMT to QAMT_0_, the fidelity of the data generated with fewer modules in QAMT shows a step-like decreasing trend. The results of the fidelity evaluation experiments clearly demonstrate that omitting the variable dependencies assurance (QAMT_4_) leads to a decline in data quality. Similarly, comparing QAMT_4_ and QAMT_3_, the lack of HKG leads to a significant performance decrease, with a more noticeable drop compared to the exclusion of variable dependencies. It shows that the external knowledge provided by the HKG helps with higher-quality medical time-series data generation. Moreover, the data generated without clinical constraints (QAMT_2_) also result in decreased fidelity performance. This is because during the data generation process, both GANs and LLMs may produce incorrect outputs. Therefore, comparing QAMT_2_ and QAMT_4_, we find that the clinical constraints applied after generation using the HKG-CoT and CKG can effectively eliminate these errors. We find that the medical time-series data generated with the simultaneous usage of LLM and GAN (QAMT_2_) have higher fidelity compared with the single usage of LLM or GAN for data generation (QAMT_1_ and QAMT_0_), showing that the modularization of the generation process in QAMT is important.

#### 8.7.2. Utility Evaluation

**Figure 7:**
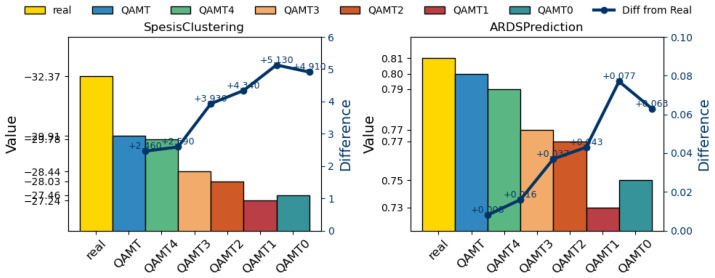
Utility metrics for medical time-series data in ablation experiments. Each point in the line chart represents the difference between ${\mathrm{QAMT}}_{n}$ and the real data.

Figure 7 demonstrates that, in downstream tasks, medical time-series data generated without applying the simultaneous usage of the LLM and GAN, HKG, clinical constraints, and variable dependencies perform worse than the data generated by QAMT. This further demonstrates the importance of our modular design in QAMT.

#### 8.7.3. Privacy Evaluation

Table 10 reports that the privacy risk of the data generated by QAMT, QAMT_4_, QAMT_3_, QAMT_2_, QAMT_1_, and QAMT_0_ show a step-like increasing trend, indicating the significance of our modular design in QAMT.

## 9. Conclusions

In this study, we proposed QAMT, an LLM-based framework for quality-assured medical time-series data generation. The framework constructs a reliable HKG to inject medical expertise into LLMs and uses a dual-module method to jointly generate medical time-series data, including static event data and temporal data. In addition, QAMT incorporates a quality assurance module to evaluate the generated data. It provides clinical constraint assurance in static event data based on an HKG-CoT and in temporal data based on a CKG and employs LLM-based prediction to ensure variable dependencies. Unlike existing methods, QAMT maintains the modularity and high-level pipeline structure of the generation process, preserving interpretability.

Currently, the proposed QAMT is only applicable to the medical domain. Other domain-specific areas, such as energy [29], also involve time-series data generation tasks. Therefore, in the future, we plan to extend QAMT to other domains to support other time-series data generation tasks.

## Figures and Tables

**Table 1:** Comparison of medical time-series data generation methods.

Method	Type	Domain	JointGeneration	QualityAssurance ^1^	Interpretability
SynTEG [20]	GAN-based	Medical	✗	-	✗
BGAN [21]	GAN-based	Medical	✗	-	✗
MTGAN [22]	GAN-based	Medical	✗	-	✗
VGAE [23]	VAE-based	Medical	✗	-	✗
PromptEHR [19]	LLM-based	Medical	✗	CC, VD	✗
CEHR-GPT [24]	LLM-based	Medical	✗	CC	✗
RTSGAN [25]	GAN-based	Medical	✗	-	✗
TimEHR [26]	GAN-based	Medical	✗	-	✗
EHR-Safe [27]	GAN-based	Medical	✗	-	✗
CodeAR [28]	VAE-based	Medical	✗	-	✗
GenG [29]	LLM-based	Energy	✗	-	✗
HGAN [16]	GAN-based	Medical	✓	CC, VD	✗
HALO [9]	LLM-based	Medical	✓	CC	✗
SynEHRgy [10]	LLM-based	Medical	✓	-	✗
QAMT (ours)	LLM-based	medical	✓	CC, VD	✓

^1^ “CC” refers to clinical constraints, and “VD” refers to variable dependencies. “✓” indicates that the method has solved the corresponding challenge, while “✗” indicates that it has not been solved.

**Table 2:** Examples of edge relationships in the HKG.

Relation	Source Node ^1,2^	Target Node ^1,2^	Example
Concept → Concept			
_has_symptom_	Dis	Sym	COPD -_has_symptom_→ fever
_is_treated_by_	Dis/Sym	Med/Tre	COPD -_is_treated_by_→ β-agonist
_indicated_by_	Dis	CI	Diabetes -_indicated_by_→ HbA1c
_progresses_to_	Dis	Dis	Prediabetes -_progresses_to_→ Type 2 Diabetes
_treated_	Med/Tre	Dis/Sym	β-agonist -_treated_→ COPD
_cause_	Sym	Dis	fever -_cause_→ COPD
_measured_	CI	Dis	PaO_2_ -_measured_→ COPD
_constraints_	CI	CC	PaO_2_ -_constraints_→ 20–50 mmHg
…			
Entity/Event → Concept			
_conforms_to_	Entity/Event	CC	EUI:67178.Lab Result -_conforms_to_→ HbA1c > 7%
_diagnoses_	Event	Dis	EUI:43613.Description -_diagnoses_→ Hypertension
_prescribes_	Event	Med/Tre	EUI:54241.Prescription -_prescribes_→ Amoxicillin
_associated_with_	Event	CI	EUI:67129.Indicators -_associated_with_→ ${\mathrm{PaO}}_{2}$
…			
Entity/Event → Entity/Event			
_participate_in_	Entity	Event	IUI:7657 -_participate_in_→ EUI:67129
_associate_with_	Entity (A/Eth)	Event	Age 65 -_associate_with_→ EUI:412546
_occurs_at_	Event	Entity (L)	EUI:63415 -_occurs_at_→ Beijing
_time_is_	Event	Entity (T)	EUI:132807 -_time_is_→ Year 2010
_event_type_is_	Event	Entity (ET)	EUI:67129 -_event_type_is_→ Surgery
_after_/_before_	Event	Event	EUI:67129 -_after_→ EUI:162308
…			

^1^ For concept nodes, “Dis” refers to diseases, “Sym” refers to symptoms, “Med” refers to medications, “Tre” refers to treatments, “CI” refers to clinical indicators, and “CC” refers to clinical constraints. ^2^ For entity nodes, “A” refers to age, “Eth” refers to ethnicity, “L” refers to location, “T” refers to time, “ET” refers to static event type.

**Table 3:** Fidelity metrics for static event data ^1^.

	MIMIC-III dataset			
	Unigram	Bigram	Trigram	Sequential Bigram
HGAN	0.832	0.445	0.513	0.487
SynEHRgy	0.907	0.717	**0.738**	0.571
HALO	0.872	0.287	0.313	0.521
SynTEG	0.858	0.501	0.647	0.562
QAMT	**0.928**	**0.721**	0.718	**0.631**
	eICU dataset			
	Unigram	Bigram	Trigram	Sequential Bigram
HGAN	0.799	0.319	0.483	0.366
SynEHRgy	0.848	**0.763**	0.711	0.500
HALO	0.769	0.293	0.281	0.474
SynTEG	0.787	0.579	0.663	0.492
QAMT	**0.897**	0.755	**0.736**	**0.592**

^1^ **Bold** and underline values indicate the best and second-best results, respectively.

**Table 4:** Fidelity metrics for temporal data ^1^.

	MIMIC-III dataset				
	Precision	Recall	Density	Coverage	$MSE_{corr}$
SynEHRgy	0.781 (0.011)	0.853 (0.003)	0.711 (0.016)	**0.852 (0.008)**	0.036
HGAN	0.731 (0.023)	0.617 (0.028)	**0.745 (0.045)**	0.315 (0.004)	0.083
HALO	0.503 (0.038)	0.461 (0.002)	0.372 (0.029)	0.215 (0.009)	0.075
SynTEG	0.610 (0.013)	0.721 (0.006)	0.672 (0.031)	0.507 (0.005)	0.045
QAMT	**0.811 (0.009)**	**0.859 (0.011)**	0.739 (0.036)	0.638 (0.003)	**0.024**
	eICU dataset				
	Precision	Recall	Density	Coverage	$MSE_{corr}$
SynEHRgy	0.814 (0.018)	**0.822 (0.005)**	0.701 (0.011)	0.714 (0.007)	0.042
HGAN	0.669 (0.037)	0.691 (0.042)	**0.728 (0.032)**	0.594 (0.004)	0.045
HALO	0.400 (0.061)	0.417 (0.024)	0.296 (0.018)	0.395 (0.013)	0.062
SynTEG	0.523 (0.020)	0.806 (0.012)	0.633 (0.022)	0.678 (0.006)	0.051
QAMT	**0.863 (0.017)**	0.817 (0.009)	0.698 (0.025)	**0.743 (0.005)**	**0.033**

^1^ **Bold** and underline values indicate the best and second-best results, respectively.

**Table 5:** Utility metrics for medical time-series data ^1^.

	MIMIC-III dataset			
	SpesisClustering (ΔSSE %)	SpesisTreatment (ΔQ)	ARDSPrediction (AUROC)	ARDSTreatment (ΔMortalityRate %)
Real data	−32.37	0.217	0.809	−2.33
HGAN	−28.67	0.189	0.764	−1.73
SynEHRgy	−28.11	0.203	0.818	−2.17
HALO	−27.72	0.195	0.793	−2.11
SynTEG	−28.02	0.191	0.799	−2.13
QAMT	**−29.91**	**0.214**	**0.801**	**−2.28**
	eICU dataset			
	SpesisClustering (ΔSSE %)	SpesisTreatment (ΔQ)	ARDSPrediction (AUROC)	ARDSTreatment (ΔMortalityRate %)
Real data	−40.82	0.172	0.813	−2.49
HGAN	−38.82	0.161	0.825	−2.01
SynEHRgy	−37.41	0.167	0.816	**−2.31**
HALO	−43.58	0.165	**0.814**	−2.16
SynTEG	−40.07	0.162	0.820	−2.20
QAMT	**−42.17**	**0.173**	0.807	−2.24

^1^ **Bold** and underline values indicate the best and second-best results, respectively.

**Table 6:** Privacy metrics for medical time-series data.

MIMIC-III Dataset								
Static Event Data					Temporal Data			
Method	JSD	WD	AUROC		Method	JSD	WD	AUROC
HGAN	0.015	0.001	0.482		HGAN	0.001	0.003	0.482
SynEHRgy	0.014	0.001	0.461		SynEHRgy	0.002	0.002	0.492
HALO	0.013	0.000	0.477		HALO	0.003	0.001	0.493
SynTEG	0.014	0.000	0.469		SynTEG	0.002	0.001	0.488
QAMT	0.013	0.000	0.456		QAMT	0.001	0.002	0.477
eICU Dataset								
Static Event Data					Temporal Data			
Method	JSD	WD	AUROC		Method	JSD	WD	AUROC
HGAN	0.015	0.002	0.496		HGAN	0.001	0.001	0.497
SynEHRgy	0.015	0.002	0.479		SynEHRgy	0.001	0.002	0.508
HALO	0.015	0.001	0.486		HALO	0.003	0.002	0.509
SynTEG	0.015	0.001	0.481		SynTEG	0.002	0.002	0.506
QAMT	0.015	0.001	0.456		QAMT	0.001	0.002	0.504

**Table 7:** Statistical significance test.

		Static event data	
		Unigram	Bigram
Value		0.928	0.721
95% CI		[0.905, 0.951]	[0.712, 0.730]
*p*		*p* < 0.05	*p* < 0.05
		Temporal data	
		Precision	Recall
Value		0.811	0.859
95% CI		[0.802, 0.820]	[0.852, 0.866]
*p*		*p* < 0.05	*p* < 0.05
		Time-series data	
		SpesisClustering (ΔSSE %)	SpesisTreatment (ΔQ)
Value		−29.91	0.214
95% CI		[−30.27, −29.55]	[0.211, 0.217]
*p*		*p* < 0.05	*p* < 0.01

**Table 8:** Noise robustness analysis.

Noise Intensity	Precision	Recall	Density	Coverage	${\mathrm{MSE}}_{\mathrm{corr}}$
0%	0.811	0.859	0.739	0.638	0.024
10%	0.806	0.848	0.735	0.636	0.025
20%	0.803	0.841	0.728	0.633	0.025
Influence	0.6%	2.1%	1.5%	0.8%	4.2%

**Table 9:** Parameter sensitivity analysis.

*k*	Precision	Recall	Density	Coverage	${\mathrm{MSE}}_{\mathrm{corr}}$
3	0.790	0.837	0.728	0.615	0.022
5	0.811	0.859	0.739	0.638	0.024
7	0.814	0.871	0.743	0.644	0.025
10	0.815	0.873	0.744	0.646	0.025

**Table 10:** Privacy metrics for medical time-series data in ablation experiments.

Static Event Data					Temporal Data			
Method	JSD	WD	AUROC		Method	JSD	WD	AUROC
QAMT	0.013	0.000	0.456		QAMT	0.001	0.002	0.477
QAMT_4_	0.016	0.004	0.457		QAMT_4_	0.002	0.002	0.478
QAMT_3_	0.044	0.013	0.462		QAMT_3_	0.005	0.006	0.484
QAMT_2_	0.050	0.014	0.465		QAMT_2_	0.005	0.007	0.486
QAMT_1_	0.071	0.019	0.470		QAMT_1_	0.006	0.008	0.491
QAMT_0_	0.068	0.018	0.469		QAMT_0_	0.006	0.008	0.492

## Data Availability

We use the open source data MIMIC-III and eICU. The data provided by the Emergency Department of Peking University People’s Hospital is unavailable due to privacy concerns.
